# Liver steatosis and metabolic dysfunction-associated fatty liver disease among HIV-positive and negative adults in urban Zambia

**DOI:** 10.1136/bmjgast-2022-000945

**Published:** 2022-07-13

**Authors:** Belinda Varaidzo Chihota, Carlotta Riebensahm, Guy Muula, Edford Sinkala, Roma Chilengi, Lloyd Mulenga, Samuel Bosomprah, Michael J Vinikoor, Carolyn Bolton-Moore, Matthias Egger, Andri Rauch, Annalisa Berzigotti, Gilles Wandeler, IeDEA-southern Africa

**Affiliations:** 1 Centre for Infectious Disease Research in Zambia, Lusaka, Zambia; 2 Institute of Social and Preventive Medicine, University of Bern, Bern, Switzerland; 3 Graduate School of Health Sciences, University of Bern, Bern, Switzerland; 4 Department of Infectious Diseases, Bern University Hospital, University of Bern, Bern, Switzerland; 5 Department of Internal Medicine, University Teaching Hospital, Lusaka, Zambia; 6 Ministry of Health, Lusaka, Zambia; 7 Department of Biostatistics, University of Ghana, Accra, Ghana; 8 Department of Medicine, The University of Alabama, Birmingham, Alabama, USA; 9 Centre for Infectious Disease Research, University of Cape Town, Cape Town, South Africa; 10 Department of Visceral Surgery and Medicine, Inselspital, Bern University Hospital, University of Bern, Bern, Switzerland

**Keywords:** FATTY LIVER, HIV/AIDS, LIVER

## Abstract

**Introduction:**

The growing importance of non-communicable diseases (NCDs) and high HIV prevalence in urban African settings may increase the burden of metabolic dysfunction-associated fatty liver disease (MAFLD). We assessed liver steatosis among HIV-positive and negative adults in urban Zambia.

**Methods:**

Adults 30 years and older who were newly diagnosed with HIV, or tested HIV-negative at two primary care clinics in Lusaka, Zambia, were assessed for liver steatosis. Cardiometabolic data were collected through comprehensive clinical and laboratory assessments. Transient elastography was performed to measure controlled-attenuation parameter (≥248 dB/m). We used multivariable logistic regression models to determine the factors associated with the presence of steatosis.

**Results:**

We enrolled 381 patients, including 154 (40%) antiretroviral therapy-naïve people living with HIV (PLWH) with a median CD4+ count of 247 cells/mm^3^ and a mean body mass index (BMI) of 23.8 kg/m^2^. Liver steatosis was observed in 10% of participants overall and was more common among HIV-negative adults than in PLWH (15% vs 3%). The proportion of patients with steatosis was 25% among obese (BMI ≥30 kg/m^2^) participants, 12% among those overweight (BMI 25–29.9 kg/m^2^), and 7% among those with a BMI <25 kg/m^2^. Among patients with a fasting glucose ≥7 mmol/L or confirmed diabetes, 57% had liver steatosis. In multivariable analyses, HIV status (adjusted odds ratio (aOR) 0.18, 95% CI 0.06 to 0.53), confirmed diabetes or elevated fasting glucose (aOR 3.92, 95% CI 1.57 to 9.78) and elevated blood pressure (aOR 2.95, 95% CI 1.34 to 6.48) were associated with steatosis. The association between BMI>25 kg/m^2^ and liver steatosis was attenuated after adjustment for potential confounders (aOR 1.96, 95% CI 0.88–4.40). Overall, 21 (9%) participants without HIV and 4 (3%) with HIV met the criteria for MAFLD. Among individuals with liver steatosis, 65% (95% CI 49% to 80%) fulfilled criteria of MAFLD, whereas 15 (39%) of them had elevated transaminases and 3 (8%) F2–F4 fibrosis.

**Conclusions:**

The prevalence of liver steatosis in this urban cohort of HIV-positive and negative adults in Zambia was low, despite a large proportion of patients with high BMI and central obesity. Our study is among the first to report data on MAFLD among adults in Africa, demonstrating that metabolic risk factors are key drivers of liver steatosis and supporting the adoption of the criteria for MAFLD in African populations.

**WHAT IS ALREADY KNOWN ON THIS TOPIC**

- The burden of liver diseases is increasing in sub-Saharan Africa. HIV infection, obesity and diabetes have been identified as key drivers for liver steatosis in European populations.

**WHAT THIS STUDY ADDS**

- We assessed liver steatosis in an urban cohort of HIV-positive and negative adults in Zambia. We are among the first to report on metabolic dysfunction-associated fatty liver disease (MAFLD) in an African population. We found liver steatosis to be strongly associated with metabolic risk factors irrespective of HIV status.

**HOW THIS STUDY MIGHT AFFECT RESEARCH, PRACTICE OR POLICY**

- The adoption of the recently endorsed MAFLD criteria is supported in African populations where metabolic factors are key drivers of liver steatosis.

## Introduction

The most important driver of liver-related mortality in sub-Saharan Africa (SSA) is chronic hepatitis B virus infection, which causes up to 45% of liver cancer deaths in the region.^1 2^ However, the relative contribution of non-communicable diseases (NCDs) to the burden of liver disease is expected to grow, given increasing urbanisation and transitioning lifestyles.^3 4^ In recent years, the trend towards obesogenic societies has been rising in Southern Africa compared with other regions in SSA. Recent data highlight the increasing prevalence of obesity and diabetes, which are important drivers of liver steatosis.^5^ In addition, the high prevalence of HIV infection, a condition associated with the presence of liver steatosis in high-income countries, may further increase the risk of metabolic liver disease in the region.^6^

Although the determinants of liver steatosis in African populations are not well understood, its prevalence was reported to be relatively low compared with other regions.^7^ However, studies published to date were mainly conducted among people with type 2 diabetes, with small sample sizes. None of the studies used controlled attenuation parameter (CAP) using transient elastography, with the ability to simultaneously measure liver stiffness.^7^ There is an urgent need for epidemiological data on the prevalence of metabolic liver disease among people living with HIV (PLWH) and on the relative impact of other cardiometabolic risk factors.

The recent adoption of criteria for characterising metabolic dysfunction-associated fatty liver disease (MAFLD) is timely, as numerous studies have shown associations of type 2 diabetes mellitus (T2DM), hypertriglyceridaemia and obesity with liver steatosis.^8^ With rising trends of metabolic-related comorbidities among PLWH and urban dwellers in Africa, it is important to establish the burden of liver steatosis in this population. Considering liver disease in relation to metabolic manifestations among HIV populations encourages implementing an integrated disease approach at the primary care level.^9^ This study aimed to describe the prevalence of liver steatosis and characterise MAFLD among treatment-naïve PLWH and HIV-negative adults attending primary care clinics in urban Zambia.

## Patients and methods

### Study design and participants

This was a cross-sectional analysis of adults aged 30 years and older enrolled into a prospective cohort study at Kalingalinga Health Centre and Matero Level 1 Hospital in urban Lusaka, Zambia, between August 2019 and June 2021. Both sites include middle-class and lower-class catchment populations. This study and both clinics are part of the International Epidemiology Databases to Evaluate AIDS collaboration in Southern Africa.^10^ We included newly diagnosed PLWH who were treatment-naïve at the point of enrolment and HIV-negative adults who presented at the same facility seeking an HIV test and had a documented negative test result.

### Clinical data collection

At enrolment, we collected comprehensive sociodemographic and clinical data using standardised questionnaires and physical examination. Blood pressure was measured using the OMRON Bronze upper arm blood pressure monitor BP510 (OmronHealthcare). Height and weight were measured using the Seca 813 scale and Seca 0123 stadiometer, respectively. Hip and waist circumference were measured using a standard metric tape measure. Alcohol consumption was assessed using the Alcohol Use Disorders Identification Test-Concise (AUDIT-C) questionnaire.^11^ We tested for hepatitis B surface antigen (HBsAg) using the Alere Determine HBsAg rapid test and for hepatitis C infection using the SD Bioline Hepatitis C virus (HCV) antibody rapid test. Serum samples were collected for alanine aminotransaminase (ALT), alkaline phosphatase (ALP) and aspartate aminotransferase (AST) measurements. Patients were required to return fasted (≥8 hours) for a second visit within 2 weeks of enrollment for glucose and lipid panels, as well as transient elastography. Blood tests were performed at the central laboratory using the COBAS Beckman Coulter chemistry analyzer and the Alere Afinion 2 analyser for lipid panels.

### Transient elastography

Transient elastography was performed at the 2-week visit in fasting patients. A trained research nurse measured liver steatosis by CAP in dB/m and liver fibrosis by liver stiffness measure (LSM in kPa) using the Echosens FibroScan 430 mini (Paris, France) with an M probe. Patients requiring an XL probe were recalled for transient elastography examination on a separate visit where further attempts were made by the research nurse. After three attempts the scan was documented as failed if XL probe was advised. Results were expressed as a median and were considered valid if ≥10 successful measurements were taken, the success rate was ≥60%, and the LSM IQR to median ratio (IQR/med) was lower than 30% of the median value.^12^

### Outcomes and definitions

Our primary outcome was liver steatosis, defined as CAP ≥248 dB/m. We further staged steatosis into categories S1 (mild steatosis) if CAP 248–267 dB/m, S2 (moderate steatosis) if CAP 268–279 dB/m and S3 (severe steatosis) if CAP≥280 dB/m.^13 14^ Significant liver fibrosis was defined according to Metavir-equivalent stages as LSM ≥7.0 kPa and cirrhosis as LSM ≥11 kPa.^15^ Elevated transaminases were defined as ALT>20 U/mL for women and >30 U/mL for men. According to reference ranges from our laboratory, ALP and AST were defined as elevated with >138 U/L and >38 U/L, respectively. Hazardous alcohol consumption was defined as AUDIT-C score ≥4 for men and ≥3 women.^16^ We diagnosed MAFLD as the presence of liver steatosis detected by CAP plus the presence of T2DM (confirmed diagnosis or antidiabetic treatment) or having a body mass index (BMI) of 25 kg/m^2^ and above. For patients with a normal or low BMI, MAFLD was present if they had at least two of the following metabolic abnormalities: waist circumference ≥102 cm for men or ≥88 cm for women, blood pressure ≥130/85 mm Hg or taking antihypertensive treatment, high-density lipoprotein cholesterol <1.0 mmol/L for men or <1.3 mmol/L for women, plasma triglycerides≥1.70 mmol/L and pre-diabetes defined as fasting plasma glucose between 5.6 and 6.9 mmol/L^8^.

### Statistical analysis

We summarised categorical variables using frequencies and proportions and used the median and IQR for continuous variables. Pearson’s χ^2^ test or Fisher’s exact test (where appropriate) were used to test for differences in baseline characteristics by HIV status. The prevalence of liver steatosis was calculated as the number of participants with CAP ≥248 dB/m divided by the total number of eligible participants. We used multivariable logistic regression to identify factors associated with liver steatosis and selected variables a priori. A p value less than 0.05 was considered statistically significant. All analyses were performed in Stata V.16 (StataCorp).

## Results

### Characteristics of participants

Of 467 patients enrolled in the cohort, 381 (82%) had complete data for this analysis, including 154 (40%) PLWH and 227 (60%) HIV-negative individuals. Patients with incomplete data included 20 (4%) who missed the second visit, 25 (5%) with unsuccessful elastography measurements, 5 (6%) for whom an XL probe was advised, 36 (8%) who were recently enrolled and still awaiting on liver assessment (online supplemental appendix 1). The median age was higher among PLWH (39 years, IQR 33–44) compared with HIV-negative adults (35 years, IQR 32–43), and there were more men among the PLWH (49% vs 33%) (table 1). Among PLWH, median CD4+ count was 247 cells/mm^3^ (IQR 104–421), and median HIV viral load was 11,071 copies/ml (IQR 88–349,189). HIV-negative adults were more likely to have a BMI≥25 kg/m^2^ (40% vs 21%), to have central obesity (31% vs 18%) and to have elevated systolic blood pressure (28% vs 19%) than PLWH. In contrast, the prevalence of other cardiovascular risk factors such as hyperglycaemia, hyperlipidaemia and elevated blood pressure was similar between groups. Overall, hazardous alcohol consumption was reported by more than half of the patients, and 12 (3%) had a positive HBsAg. No participants tested positive for HCV antibodies.

10.1136/bmjgast-2022-000945.supp1 Supplementary data

**Table 1:** Demographic and clinical characteristics of participants, by HIV status

Characteristics	HIV-positive (n=154)	HIV-negative (n=227)	P value
Median age, years (IQR)	39 (33–44)	35 (32–43)	***<*0.01**
Male sex, n (%)	76 (49)	76 (33)	***<*0.01**
Marital status, n (%)			***<*0.01**
Married	78 (50)	144 (63)	
Divorced/separated/widowed	58 (38)	52 (23)	
Single	18 (12)	31 (14)	
Employment, n (%)			***<*0.01**
Employed/self-employed	116 (75)	130 (58)	
Unemployed	38 (25)	96 (42)	
Alcohol consumption, n (%)			0.45
Abstinent	72 (47)	97 (43)	
Moderate	6 (4)	15 (6)	
Hazardous (AUDIT-C ≥3 for women; ≥4 for men)	76 (49)	117 (51)	
Clinical characteristics			
Body mass index, n (%)			***<*0.01**
Underweight (<18 kg/m^2^)	22 (14)	18 (8)	
Normal (18–24.9 kg/m^2^)	100 (65)	119 (52)	
Overweight (25–29.9 kg/m^2^)	22 (14)	47 (21)	
Obese (≥30 kg/m^2^)	10 (7)	43 (19)	
Waist circumference, n (%)			***<*0.01**
Normal	125 (82)	156 (69)	
Abdominal obesity (≥88 cm for women; ≥102 for men)	28 (18)	71 (31)	
Missing	1	–	
Systolic blood pressure, n (%)			**0.03**
Normal	117 (76)	149 (66)	
Elevated (≥130 mm Hg)	37 (24)	78 (34)	
Diastolic blood pressure, n (%)			0.54
Normal	107 (69)	151 (66)	
Elevated (≥85 mm Hg)	47 (31)	76 (33)	
Fasting glucose, n (%)			0.51
Normal (<5.6 mmol/L)	130 (86)	202 (89)	
Pre-diabetes (5.6–6.9 mmol/L)	19 (11)	20 (8)	
Diabetes (>7 mmol/L)	3 (2)	4 (2)	
Missing	2 (1)	1 (1)	
Total cholesterol, n (%)			0.27
Normal	133 (89)	192 (85)	
Elevated (>5 mmol/L)	16 (10)	33 (14)	
Missing	5 (1)	2 (1)	
Triglycerides, n (%)			0.33
Normal	132 (86)	205 (91)	
Elevated (≥1.70 mmol/L)	18 (12)	20 (8)	
Missing	4 (3)	2 (1)	
HDL-cholesterol, n (%)			***<*0.01**
Normal	60 (39)	150 (66)	
Reduced (<1.0 mmol/L for men or <1.3 mmol/L)	90 (58)	75 (33)	
Missing	4 (3)	2 (1)	
Hepatitis B surface antigen, n (%)			0.09
Positive	2 (1)	10 (4)	
Negative	152 (99)	217 (96)	

Significant P-value in bold (p<0.05).

AUDIT-C, Alcohol Use Disorders Identification Test-Concise; HDL, high-density lipoprotein.

### Factors associated with liver steatosis

**Figure 1:**
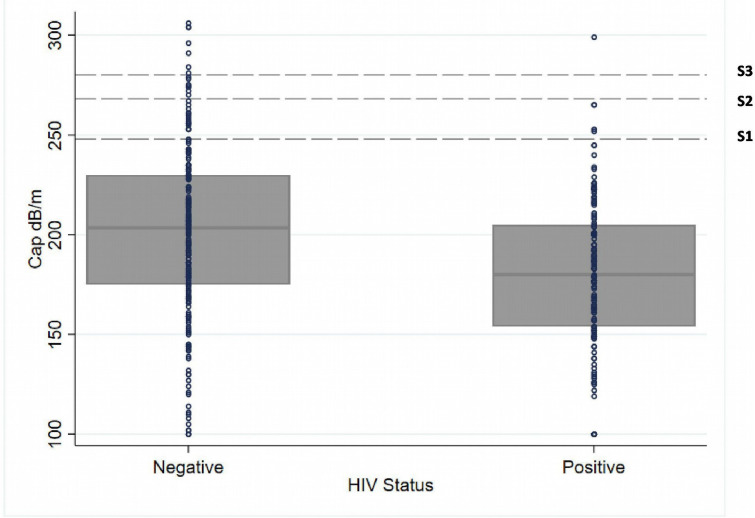
Box plot of liver steatosis values measured by continued attenuation parameter (CAP), by HIV status.

Thirty-eight of 381 adults (10%) had liver steatosis, and 8 (2%) had severe steatosis (figure 1, table 2). The prevalence of liver steatosis was 15% (33/227) among HIV-negative adults, and 3% (5/154) in PLWH (table 2). The percentage of patients with steatosis was 25% among obese (BMI ≥30 kg/m^2^) participants, 12% among those overweight (BMI 25–30 kg/m^2^) and 7% among those with a BMI <25 kg/m^2^. The percentage of individuals with liver steatosis was 57% among patients with fasting glucose ≥7 mmol/L or confirmed diabetes, 25% among pre-diabetes (fasting glucose: 5.6–6.9 mmol/L) and 8% among those with normal blood glucose. The prevalence of liver steatosis was the same in the groups of patients with or without hazardous alcohol consumption. In multivariable analyses, HIV status (adjusted OR (aOR) 0.18, 95% CI 0.06 to 0.53), confirmed diabetes or elevated fasting glucose (aOR 3.92, 95% CI 1.57 to 9.78) and elevated blood pressure (aOR 2.95, 95% CI 1.34 to 6.48) were associated with steatosis (table 3). There was no clear association of liver steatosis with male sex (aOR 1.40, 95% CI 0.59 to 3.38), age >40 years (aOR 1.85, 95% CI 0.84 to 4.05) or hazardous alcohol consumption (aOR 1.11, 95% CI 0.49 to 2.47).

**Table 2:** Liver-related characteristics, by HIV status

Characteristics	HIV-positive (n=154)	HIV-negative (n=227)	Total (n=381)
Liver steatosis, n (%)			
Yes	5 (3)	33 (15)	38 (10)
No	149 (97)	194 (85)	343 (90)
Steatosis staging, n (%)			
S0 (CAP <248 dB/m)	149 (96)	194 (85)	343 (90)
S1 (CAP 248–267 dB/m)	4 (3)	18 (8)	22 (6)
S2 (CAP 268–279 dB/m)	0	8 (4)	8 (2)
S3 (CAP ≥280)	1 (1)	7 (3)	8 (2)
Fibrosis/cirrhosis, n (%)			
Yes	14 (9)	18 (8)	32 (8)
No	140 (91)	209 (92)	349 (92)
Fibrosis staging, n (%)			
Normal	140 (91)	209 (92)	349 (92)
Fibrosis (LSM ≥7 kPa)	11 (7)	13 (6)	24 (6)
Cirrhosis (LSM≥11 kPa)	3 (2)	5 (2)	8 (2)
ALT, median (IQR)	24 (17–36)	19 (15–29)	21 (16–31)
ALT, n (%)			
Normal	79 (51)	143 (63)	222 (58)
Elevated (>20 U/mL for women; >30 U/mL for men)	75 (49)	84 (37)	159 (42)
AST, median (IQR)	36 (26–49)	25 (20–32)	28 (22–39)
AST, n (%)			
Normal	86 (56)	190 (84)	276 (72)
Elevated (>38 U/L)	66 (43)	33 (15)	99 (26)
Missing	2 (1)	4 (1)	6 (2)
ALP, median	79 (65–105)	70 (57–85)	73 (59–89)
ALP, n (%)			
Normal	140 (91)	217 (96)	357 (94)
Elevated (138 U/L)	10 (6)	6 (3)	16 (4)
Missing	4 (3)	4 (1)	8 (2)

ALP, alkaline phosphatase; ALT, alanine aminotransaminase; AST, aspartate aminotransferase; CAP, controlled-attenuation parameter; LSM, liver stiffness measure.

**Table 3:** Factors associated with liver steatosis

	Steatosis S1–S3	Univariable analysis		*Multivariable analysis	
	n/total no (%)	OR (95% CI)	P value	OR (95% CI)	P value
HIV status					
Negative	33/227 (15)	1		1	
Positive	5/154 (3)	0.20 (0.08 to 0.52)	*<*0.001	0.18 (0.06 to 0.53)	**0.002**
Sex					
Female	22/229 (10)	1		1	
Male	16/152 (11)	1.11 (0.56 to 2.18)	0.77	1.40 (0.59 to 3.38)	0.44
Age group, years					
30–40	19/239 (8)	1		1	
40+	19/142 (13)	1.79 (0.91 to 3.51)	0.09	1.85 (0.84 to 4.05)	0.12
BMI					
<25 kg/m^2^	17/259 (7)	1		1	
≥25 kg/m^2^	21/122 (17)	2.96 (1.49 to 5.84)	0.002	1.96 (0.88 to 4.40)	0.1
Fasting glucose					
Normal	26/332 (8)	1	*<*0.001	1	
Elevated/treatment	12/46 (26)	4.15 (1.92 to 8.97)		3.92 (1.57 to 9.78)	**0.007**
Missing	0/3				
Blood pressure					
Normal	16/288 (6)	1		1	
Elevated/treatment	22/93 (24)	5.26 (2.63 to 10.6)	*<*0.001	2.95 (1.34 to 6.48)	**0.007**
Triglycerides					
Normal	32/337 (10)	1		1	
Elevated/treatment	6/38 (16)	1.79 (0.69 to 4.60)	0.23	0.94 (0.31 to 2.84)	0.9
Missing	0/6				
Alcohol consumption					
Abstinent/moderate	18/190 (10)	1		1	
Hazardous	20/191 (10)	1.11 (0.57 to 2.19)	0.76	1.19 (0.54 to 2.65)	0.8

Significant P-value in bold (p<0.05).

*Complete-case analysis (n=375).

BMI, body mass index.

Although there were no associations between alcohol consumption and steatosis the proportion of patients with hazardous alcohol consumption and steatosis was 53% (20/38). The association between BMI>25 kg/m^2^ and liver steatosis was attenuated after adjustment for potential confounders (aOR 1.96, 95% CI 0.88 to 4.40).

### Metabolic-associated fatty liver disease

**Figure 2:**
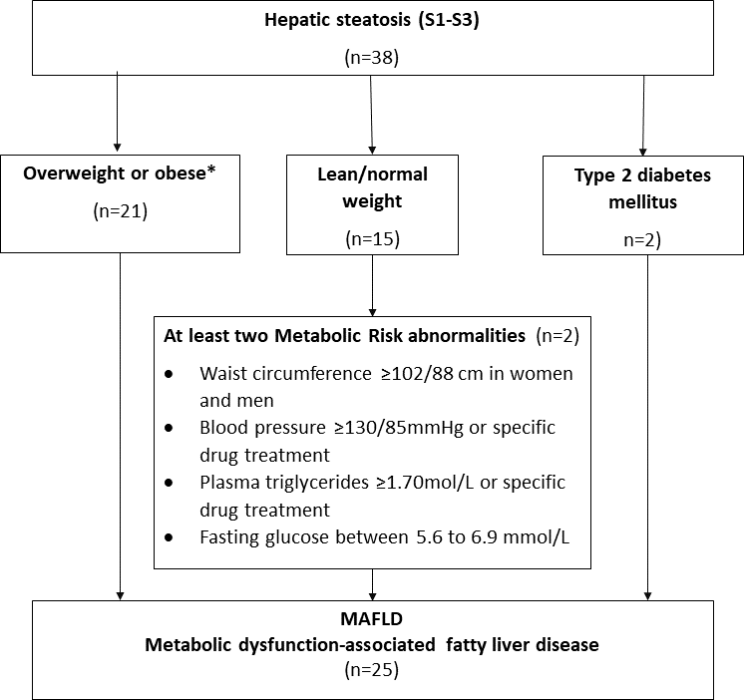
Flow chart for MAFLD diagnosis among study participants in urban Zambia. MAFLD (metabolic dysfunction-associated fatty liver disease). *2 patients with T2DM and BMI>25 kg/m2 simultaneously fulfilling MAFLD criteria through the independent pathway of being overweight/obese.

Overall, 4/154 (2.6%) PLWH and 21/227 (9.3%) persons without HIV infection had MAFLD. Among those with steatosis, 21 (64%) HIV-negative individuals and 4 (80%) PLWH had MAFLD (figure 2). Sixty-five per cent of those with liver steatosis met the criteria for MAFLD. Among the 38 individuals with liver steatosis, 13 did not fulfil the criteria for MAFLD: one among PLWH and 12 among HIV-negative individuals. Of 15 patients with liver steatosis and normal BMI, only 2 had at least two metabolic risk factors and qualified for MAFLD.

### Liver fibrosis, cirrhosis and inflammation

The percentage of individuals with liver fibrosis or cirrhosis was 9% among PLWH and 8% in HIV-negative participants, whereas ALT elevation was more common among PLWH (49% vs 37%, table 2). Among patients with steatosis, 15 (39%) had elevated transaminases, and 3 (8%) had F2-F4 fibrosis. Two HIV-negative patients had steatosis, elevated transaminases and F2–F4. The first was a male aged 32 years with cirrhosis (LSM: 13.9 kPa), S3 (CAP: 304 dB/m), BMI of 44 kg/m^2^, elevated blood pressure (189/109 mm Hg) and hazardous alcohol consumption. The second was a female aged 42 years with F3 (LSM: 9.1 kPa), S1 (CAP: 263 dB/m), normal BMI of 18.7 kg/m^2^, elevated blood pressure (132/88 mm Hg) and hazardous alcohol consumption.

## Discussion

We found a relatively low prevalence of liver steatosis in this cohort of HIV-positive and negative adults ≥30 years in urban Zambia. Despite the high proportion of individuals with elevated BMI and central obesity, only 25% of those with BMI ≥30 kg/m^2^ and 12% among those with BMI 25–30 kg/m^2^ had liver steatosis. MAFLD was considerably more frequent in PLWH than in HIV-negative individuals and was nearly absent in lean, non-diabetic participants. Our study is among the first to report the determinants of MAFLD in HIV-positive and negative individuals from an urban African population. With the growing burden of NCDs within this region, our data support the inclusion of multiple liver and metabolic risk factors screening for primary healthcare in Africa.^17 18^

Compared with estimates from large studies within the general population in Europe and North America, the prevalence of liver steatosis in our cohort was low.^19–21^ This finding was surprising, given the high proportion of overweight participants in our study. Furthermore, few patients had severe steatosis, in contrast to other population-based studies in the UK where nearly half (48.3%) were diagnosed with severe steatosis.^20^ Individuals with known diabetes or elevated fasting glucose measurements were five times more likely to have liver steatosis than other participants. The prevalence of liver steatosis was 30% among people with diabetes, slightly lower than in most studies of patients with T2DM in Africa.

Studies from Sudan, Nigeria and Ethiopia reported estimates between 50% and 73%, but sample sizes were small,^22–24^ and steatosis was generally measured with ultrasound, which has a lower sensitivity and specificity for detecting liver steatosis when compared with CAP measured by transient elastography.^25^ We also found a significant association between elevated blood pressure and liver steatosis. Although this association has not been reported for SSA, reports from a large cohort in Italy and the third generation Framingham heart study in the USA showed an association between arterial hypertension and liver steatosis.^26 27^ The relationship between hypertension and liver steatosis could reflect metabolic syndrome and cardiovascular disease risk, which are known bidirectional predictors of fatty liver.^28^

Many large studies from high-income countries have reported a strong association between obesity and liver steatosis.^20 29^ Despite a higher prevalence of steatosis among obese patients in our study, most of those who were obese did not have liver steatosis. Although we found reasonable evidence of an association between BMI and liver steatosis in multivariable analyses, it was not statistically significant. Potential explanations for this finding could be our limited sample size or our dichotomisation of BMI at cut-offs that may not be adapted for African populations. Indeed, the magnitude of the point estimate observed (OR: 1.96) was lower than the estimates shown in previous studies from high-income countries. Sociocultural differences related to nutrition and unmeasured genetic differences may be potential explanations for the low prevalence of steatosis observed among obese Africans in our study.^30^ Along the same lines, we did not find any associations between cholesterol or triglycerides levels and the presence of liver steatosis, which has been reported in other populations.^31^ These observations, as well as the absence of an association between the high alcohol consumption and liver steatosis in our study population underline the potential differences in the relationship between metabolic factors and liver steatosis between people in SSA and high-income countries.^32^ The diagnosis of MAFLD does not exclude patients with alcohol consumption, however, the dual burden of alcohol and steatosis on liver diseases remains an important point of discussion.^8^

The prevalence of liver steatosis among PLWH in our study was especially low. Several studies have shown that HIV per se may not be a key driver of liver steatosis among PLWH: reports from the USA and Canada did not show a significant association between HIV and liver steatosis after adjustment for confounders.^33–35^ Instead, HIV-associated comorbidities and specific antiretroviral therapy (ART) components seem to impact the development of liver steatosis: in a recent meta-analysis of 10 published studies among PLWH, the most important predictors of liver steatosis were classical metabolic risk factors such as high BMI, waist circumference, type 2 diabetes, and hypertension.^27^ However, given the paucity of data on the prevalence of metabolic liver disease among ART-naïve individuals, our results will need to be confirmed in larger studies which include individuals with less advanced stage of disease at ART initiation. Given the current roll out of dolutegravir and tenofovir alafenamide across Africa, the measurement of liver steatosis will be of high priority, as these drugs have been associated with weight gain and lipid dysregulation.^36–38^

In our analysis, the majority of steatosis cases were seen among overweight and diabetic individuals. Of those with liver steatosis and a normal BMI, only a minority had additional metabolic risk factors as per the definition of MAFLD. Thus, lean MAFLD was almost absent in our study, in contrast to studies from high-income countries. The inclusion of these metabolic parameters for the definition of MAFLD proved to have clinical utility in a Sri Lankan cohort where this new definition captured high-risk patients who were initially excluded by the NAFLD definition.^9 39^

Our study’s rather small sample size was an important limitation: due to the SARS-CoV-2 pandemic and various lockdowns, study recruitment was irregular with various occasions when the study had to be suspended. Consequently, we could only enrol a relatively small number of PLWH, of whom many were in advanced stages of HIV infection at the time of ART initiation. More data are needed from patients in long-term care on ART in African primary care clinics to monitor potential metabolic side effects of newer ART regimens. Prospective data collection from our ongoing cohort will be crucial to understand the dynamics of liver related complications among the ageing PLWH and the general HIV-negative population from Southern Africa We could not confirm diabetes in a few patients, which could have underestimated the association with steatosis. BMI and central obesity thresholds applied were not specific to our population, but rather we used cut-offs derived from a Caucasian population. The need for more ethnic-specific criteria is essential in defining cardiometabolic parameters in our population.

Despite large numbers of patients with high BMI and central obesity in our study population, we report a low prevalence of steatosis. Our study is the first to have assessed the determinants of MAFLD among an urban cohort of adults with and without HIV in Southern Africa. Our data support the adoption of the positive criteria for MAFLD as we show that several metabolic risk factors are strongly associated with liver steatosis in this population. Future analyses will have to focus on PLWH on modern ART regimens, and more research is needed to determine thresholds for specific metabolic parameters adapted to the African setting.

## Data Availability

Data are available on reasonable request.
