# Supplementary figures and images for: Liver steatosis and metabolic dysfunction-associated fatty liver disease among HIV-positive and negative adults in urban Zambia
Source: BMJ Open Gastroenterol. 2022 Jul 13;9(1):e000945. doi: 10.1136/bmjgast-2022-000945 (PMC9280874; doi:10.1136/bmjgast-2022-000945)

**Appendix 1: STROBE Flow Chart of Study Participants**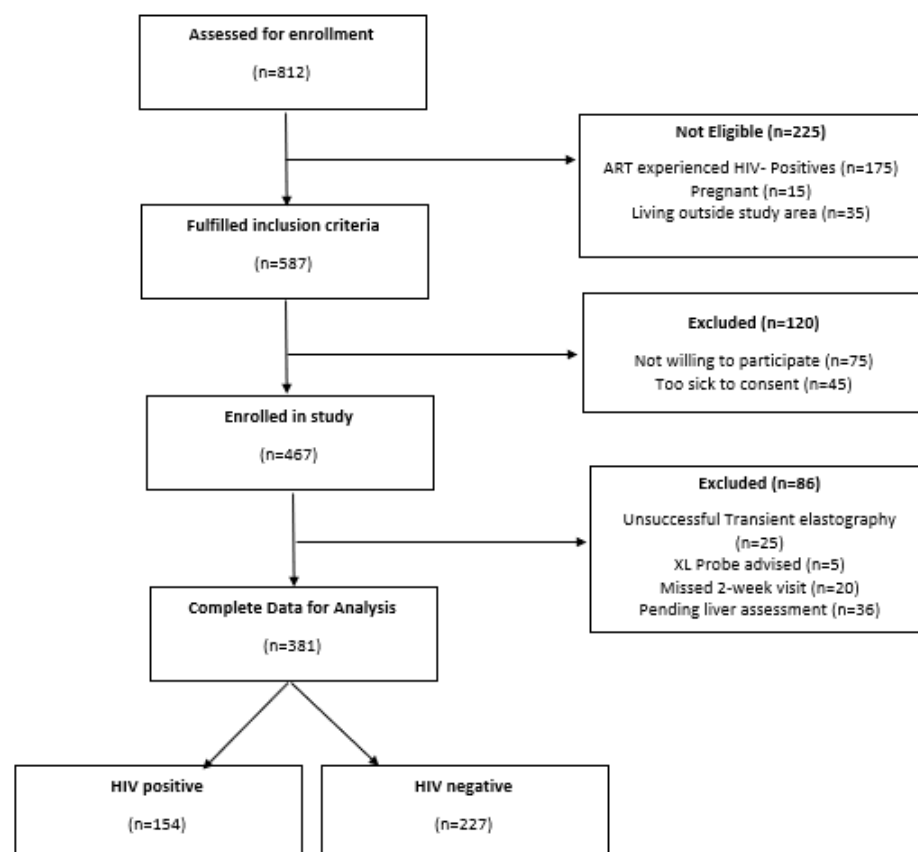

Supplement: Supplementary data [file bmjgast-2022-000945supp001.pdf]
